# Retraction Note to: Substrates specificity of tannase from *Streptomyces sviceus* and *Lactobacillus plantarum*

**DOI:** 10.1186/s13568-019-0778-5

**Published:** 2019-04-19

**Authors:** Dan Wang, Yao Liu, Die Lv, Xueli Hu, Qiumei Zhong, Ye Zhao, Mingbo Wu

**Affiliations:** 1 School of Bioscience and Biotechnology, Chengdu Medical College, 783 Xindu Avenue, Chengdu, 610500 People’s Republic of China; 2 Laboratory of Veterinary, Drug Residue Prevention and Control Technology of Animal-derived Food, Chengdu Medical College, 783 Xindu Avenue, Chengdu, 610500 People’s Republic of China

## Retraction to: AMB Expr (2018) 8:147 10.1186/s13568-018-0677-1

The Editor-in-Chief has retracted this article (Wang et al. 2018) because the authors do not have ownership of the data they report. An investigation by the Commonwealth Scientific and Industrial Research Organisation (CSIRO) has concluded that the data reported in this article are the sole property of the CSIRO. Mingbo Wu agrees with this retraction. Dan Wang, Yao Liu, Die Lv, Xueli Hu, Qiumei Zhong and Ye Zhao have not responded to correspondence about this retraction.
